# Arabidopsis Growth-Promotion and Root Architecture Responses to the Beneficial Rhizobacterium *Phyllobacterium brassicacearum* Strain STM196 Are Independent of the Nitrate Assimilatory Pathway

**DOI:** 10.3390/plants11010128

**Published:** 2022-01-04

**Authors:** Maya Kechid, Guilhem Desbrosses, Lydia Gamet, Loren Castaings, Fabrice Varoquaux, Abdelhamid Djekoun, Bruno Touraine

**Affiliations:** 1 BPMP, Université de Montpellier, CNRS, INRAE, L’Institut Agro, 34095 Montpellier, France; maya.kechid@umc.edu.dz (M.K.); guilhem.desbrosses@umontpellier.fr (G.D.); lydia.gamet@umontpellier.fr (L.G.); loren.castaings@umontpellier.fr (L.C.); 2 Laboratoire de Génétique, Biochimie et Biotechnologie Végétale (GBBV), Université Frères Mentouri-Constantine1, Route de Ain El Bey, Constantine 25000, Algeria; djak2591@gmail.com; 3 AGAP Institut, Université de Montpellier, CIRAD, INRAE, L’Institut Agro, 34095 Montpellier, France; fabrice.varoquaux@umontpellier.fr

**Keywords:** *Arabidopsis thaliana*, plant growth-promoting rhizobacteria, nitrate reductase, N metabolism, growth regulation, root architecture, *NRT2.5*, *NRT2.6*

## Abstract

*Phyllobacterium brassicacearum* STM196, a plant growth-promoting rhizobacterium isolated from roots of oilseed rape, stimulates *Arabidopsis* growth. We have previously shown that the *NRT2.5* and *NRT2.6* genes are required for this growth promotion response. Since these genes are members of the *NRT2* family of nitrate transporters, the nitrogen assimilatory pathway could be involved in growth promotion by STM196. We address this hypothesis using two nitrate reductase mutants, G5 deleted in the major nitrate reductase gene *NIA2* and G′4-3 altered in both *NIA1* and *NIA2* genes. Both mutants had a reduced growth rate and STM196 failed to increase their biomass production on a medium containing NO_3_^−^ as the sole nitrogen source. However, they both displayed similar growth promotion by STM196 when grown on an NH_4_^+^ medium. STM196 was able to stimulate lateral roots development of the mutants under both nutrition conditions. Altogether, our results indicate that the nitrate assimilatory metabolism is not a primary target of STM196 interaction and is not involved in the root developmental response. The *NIA1* transcript level was reduced in the shoots of *nrt2.5* and *nrt2.6* mutants suggesting a role for this nitrate reductase isoform independently from its role in nitrate assimilation.

## 1. Introduction

Plant roots develop associative symbioses with bacteria that colonize the rhizosphere with beneficial effects on plant growth and health which are, therefore, collectively called plant growth-promoting rhizobacteria (PGPR). Many bacterial species, from various genera, can interact with roots of a given plant species and, conversely, each PGPR strain can interact with numerous host plants, indicating a very weak host-specificity [1,2,3]. The beneficial effects of PGPR on plants are likely to be a complex response of multifactorial origin, including biofertilization, phytostimulation, and biocontrol [4,5].

A first hypothesis to explain growth promotion by PGPR consider that they enhance plant nutrition. Biofertilization can be a consequence of an increase in nutrient availability, e.g., through dinitrogen (N_2_) fixation [6], phosphate solubilization [7,8], or iron-chelating phytosiderophore production [9]. In experiments performed in vitro, these bacterial activities cannot, however, account for their plant growth-promoting effect. Firstly, the conditions are not favorable to N_2_ fixation (aerobic conditions, presence of high nitrate (NO_3_^−^) concentrations in the media) and many PGPR strains have no N_2_ fixing capacity. Secondly, phosphate and iron, as well as all the other macro- and micro-elements, are present in soluble forms and in concentrations large enough to avoid any nutritional restriction. PGPR could still have a biofertilization effect in such conditions through stimulation of root ion transport activities. For instance, *Bacillus subtilis* GB03 enhanced iron uptake and increased iron content in *Arabidopsis* through transcriptional up-regulation of *FRO2* and *IRT1* coding for a Fe^3+^ chelate reductase and a Fe^2+^ transporter, respectively [10]. In a field experiment, it was reported that a PGPR inoculum composed of different *Bacillus* species induced an upregulation of *Pht2* and *PT2-1* phosphate transporter genes in durum wheat supplied with organic fertilizer, but a downregulation of *Pht2* in unfertilized conditions [11]. Lee et al. [12] recently showed that a 4-d treatment with the *Bacillus subtilis* strain L1 strongly increased the expression of several *NRT2* genes in *Arabidopsis* including *NTR2.1*, which codes for the main high-affinity transporter involved in NO_3_^−^ uptake [13]. Although no NO_3_^−^ uptake measurement has been done in this study, this suggests that *B. subtilis* L1 could stimulate NO_3_^−^ uptake rate by the plant roots and promote N nutrition. Consistently with this hypothesis, nitrate reductase activity was increased 4-fold in *B. subtilis* L1 treated seedlings [12]. By contrast, in another study, no increase in the transcript levels of root-expressed NO_3_^−^ transporter genes were found upon inoculation of *Arabidopsis* with the canola effective PGPR strain *Phyllobacterium brassicacearum* STM196 for 8 d [14]. In this latter study, NO_3_^−^ influx was weakly increased 24-h after inoculation with STM196, but markedly decreased 8-d after inoculation. Hence, although *P. brassicacearum* STM196 did stimulate plant growth rate, no indication of a positive effect of this strain on N nutrition was found.

Alternatively, a second hypothesis to explain growth promotion by PGPR considers that the increased root surface area in PGPR inoculated plants (phytostimulation) would support a higher nutrient uptake capacity at the whole root system level, which in turn would account for increased shoot biomass production. In this hypothesis, PGPR would primarily affect root development, especially due to their ability to synthesize auxin [15,16,17] or to modify the distribution of plant-originated auxin within root tissues [18,19,20].

Based upon the occurrence of regulatory processes that adjust nutrient uptake to growth requirement [21,22], a third alternative hypothesis, rarely discussed, would be that PGPR essentially affects the root and shoot developmental programs, rather than nutritional processes or specifically the root surface involved in nutrient uptake and that higher ions uptake would only be a consequence of increased nutritional demand in faster-growing plants.

These three hypotheses are extremely difficult to disentangle because (i) nutrient uptake depends on both ion flux across the plasma membrane of absorbing cells (transporters activity) and root surface, these two parameters being usually negatively related to each other [23], and (ii) increased nutrition rate would enhance growth rate just like increased growth rate would drain higher nutrient uptake [24].

The *P. brassicacearum* STM196 strain has been proved to be a good model to address the relation between growth promotion and NO_3_^−^ nutrition in *Arabidopsis*. Firstly, it has been isolated from the rhizosphere of field-grown canola [25], a crop of the Brassicaceae family. Secondly, among the strains isolated in this study, which include also bacteria of the *Pseudomonas*, *Variovorax,* and *Agrobacterium* genera, it was the most efficient to promote the growth of canola plants, and we showed that its growth-promotion capacity is conserved in the model plant *Arabidopsis thaliana* [14]. Thirdly, in our previous studies, we obtained indications on possible interactions between the growth and root development effects of STM196 on one hand and NO_3_^−^ nutrition on the other hand [14,24,26]. Indeed, the stimulation of lateral root development was antagonistic to lateral root growth inhibition by high external NO_3_^−^ in *Arabidopsis* seedlings [14]. Therefore, STM196 is able to alleviate the nitrate-dependent control of root system architecture classically described in axenic conditions [27]. Interestingly, STM196 elicits a strong induction of *NRT2.5* and *NRT2.6* genes predominantly expressed in shoots [14]. These two genes are functionally required for growth promotion and root architecture responses to STM196 [26]. In *Arabidopsis*, the *NRT2* gene family contains 7 genes that code for NO_3_^−^ transporters. The NO_3_^−^ transport capacity of the products of all *NRT2* genes has been demonstrated using transient expression studies in *Xenopus* oocytes [28]. In addition, the NRT2s proteins, except NRT2.7, have been localized at the plasma membrane of protoplasts transformed with yellow-fluorescent protein (YFP)-labeled *NRT2* genes [28]. Taken together, it is indicating that NRT2.5 and NRT2.6 are likely NO_3_^−^ influx transporters comparable to the well-described NRT2.1. Gene expression studies provide some clues about the function of NRT2.5 and NRT2.6 specifically. Expression of *NRT2.5* is strongly induced in roots after long-term starvation and plays a role in NO_3_^−^ acquisition in such conditions [29]. However, its expression in roots is very much reduced under steady-state NO_3_^−^ nutritive conditions [14,26], so that the NRT2.5 transporter is very unlikely to significantly participate in NO_3_^−^ acquisition by the plant. In the shoots, *NRT2.5* is expressed in minor veins and the encoded transporter could be involved in the phloem loading of NO_3_^−^ [29]. As for *NRT2.6*, its transcripts are accumulated in the leaves of *Arabidopsis* inoculated with the pathogenic bacterium *Erwinia amylovora* [30]. *NRT2.6* expression, therefore, is induced in *Arabidopsis* leaves in response to both a leaf pathogenic bacterium and a root symbiotic rhizobacterium [14,26], consistent with the general scheme of partially common programs for symbiosis and disease in plants [31,32,33].

Overall, the very few data available on the physiological functions of NRT2.5 and NRT2.6 do not shed light on the role of these proteins in *Arabidopsis* responses to STM196. Since both NRT2.5 and NRT2.6 are likely to function as NO_3_^−^ transporters in planta, it is tempting to propose that STM196 triggers plant growth promotion by altering NO_3_^−^ fluxes within the plant and consequently NO_3_^−^ availability for N assimilatory metabolism. Although our previous studies did not conclude in favor of this hypothesis [14,26], the possible involvement of NO_3_^−^ metabolism rate and regulation in the plant growth promotion by STM196 cannot be ruled out.

In the present study, we investigated this latter possibility using mutants of *Arabidopsis* that are deficient either in the two nitrate reductase isoforms (NIA1 and NIA2) or in the predominant one only (NIA2). Our results demonstrate that the lateral root development response to inoculation with STM196 is independent of nitrate reductase activity, and, therefore, from NO_3_^−^ pools changes triggered by nitrate reductase mutations. Conversely, STM196 inoculation induced no significant change in nitrate reductase activity contrary to *B. subtilis* L1 strain [12]. Therefore, STM196 is unlikely to have a direct effect on N metabolism. Altogether, we provide herein evidence that STM196 triggers changes in root development and promotes plant growth, and that NO_3_^−^ endogenous pools and/or N nutrition are not responsible for these responses.

## 2. Results

### 2.1. Growth Promotion by STM196 Is Independent of NRA and Nitrate Accumulation

**Figure 1:**
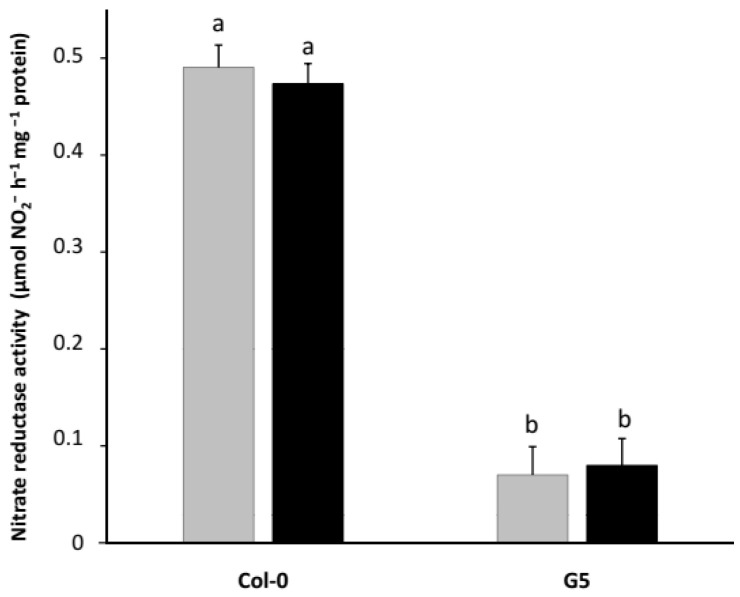
Effect of *nia2* mutation on in vitro nitrate reductase activity. *Arabidopsis* Col-0 and G5 mutant (impaired in the NIA2 major isoform of nitrate-reductase) seedlings were grown on a mineral medium containing 2 mM NO_3_^−^ as the unique N source in vertically oriented Petri dishes for 7 d, and subsequently transferred into new Petri dishes containing the same medium inoculated or not with 10^8^ cfu·mL^−1^ STM196 for 8 d. The shoots were then harvested and weighed, and in vitro nitrate reductase activity was measured as described in Materials and methods. Grey bars, seedlings grown on uninoculated media; black bars, seedlings grown on media inoculated with STM196. Data are the means ± standard deviation (*n* = 20). Different letters represent significant differences using two-way ANOVA with Fisher’s LSD multiple comparison post-test at *p* = 0.001.

Nitrate reductase activity (NRA) was hardly detectable in the roots of our young Col-0 seedlings (data not shown) while it was easily measurable in their shoots, indicating that NO_3_^−^ reduction occurs mainly in the shoots of *Arabidopsis* plants consistently with previous reports [34,35]. First, we observed that, in our growth conditions, the rhizobacterial strain *Phyllobacterium brassicacearum* STM196 did not affect shoot NRA (Figure 1). Growth promotion, therefore, cannot be a consequence of the increased NO_3_^−^ assimilation rate. However, it cannot be excluded that NRA could be involved, if not responsible, in the plant response to STM196. To address this question, conversely, we analyzed the impact of altered NRA on growth promotion by STM196. For this purpose, we used two nitrate reductase deficient mutants: the G5 mutant, which is knocked out in the *NIA2* gene coding for the major nitrate reductase isoform, and the G′4-3 mutant, which is altered in the two nitrate reductase isoforms [36,37]. Shoot NRA is 7 times lower in G5 seedlings fed with 2 mM NO_3_^−^ than in Col-0 wild-type ones grown on the same medium (Figure 1) which confirms that the NIA2 protein is responsible for the majority of nitrate reduction in *Arabidopsis*. As expected, no detectable NRA was found in the shoots of NO_3_^−^ fed G′4-3 double mutant (data not shown).

**Figure 2:**
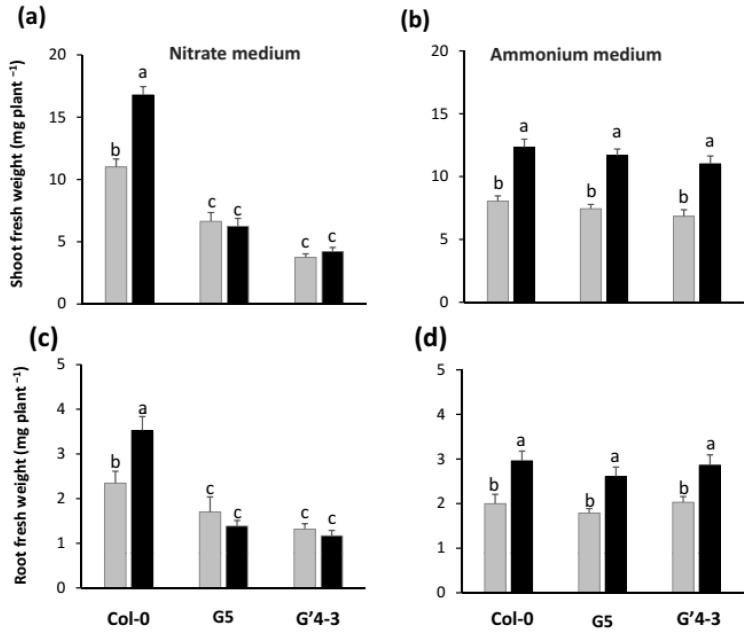
Effect of *nia2* single mutation and *nia1 nia2* double mutation on the shoot and root growth in nitrate-grown *Arabidopsis*. Seedlings of *Arabidopsis thaliana* Col-0, G5 mutant (impaired in the NIA2 major isoform of nitrate-reductase), and G′4-3 mutant (impaired in the two isoforms of nitrate-reductase) were grown on a mineral medium containing either 2 mM NO_3_^−^ or 2 mM NH_4_^+^ as the unique N source in vertically oriented Petri dishes for 7 d, and subsequently transferred into new Petri dishes containing the same media inoculated or not with 10^8^ cfu·mL^−1^ STM196 for 8 d. Shoots and roots were then harvested separately and weighed: fresh weight of the shoots of (**a**) nitrate-grown or (**b**) ammonium-grown seedlings; fresh weight of the roots of (**c**) nitrate-grown or (**d**) ammonium-grown seedlings. Grey bars, seedlings grown on uninoculated media; black bars, seedlings grown on media inoculated with STM196. Data are the means ± standard deviation (*n* = 20). Different letters represent significant differences using two-way ANOVA with Fisher’s LSD multiple comparison post-test at *p* = 0.001.

In axenic and NO_3_^−^ nutrition conditions, shoot biomass production was lowered by 38% and 66% in the G5 and G′4-3 mutants compared to the Col-0 seedlings respectively (Figure 2a). Root growth was also reduced although at a lower extent (28% and 44% in G5 and G′4-3 mutants respectively, Figure 2c). Consistently with our previous results [14,19,26], growing seedlings with roots in direct contact on a medium uniformly inoculated with STM196 at 10^8^ cfu·mL^−1^ in vertically arranged Petri dishes (for representative images of plates, see Figure S1) led to an increase of both root and shoot biomass production (Figure 2a,c). This growth promotion was abolished in both shoots and roots of G5 and G′4-3 seedlings grown on NO_3_^−^ medium (Figure 2a,c) which might indicate a role of NRA in plant response to STM196.

**Figure 3:**
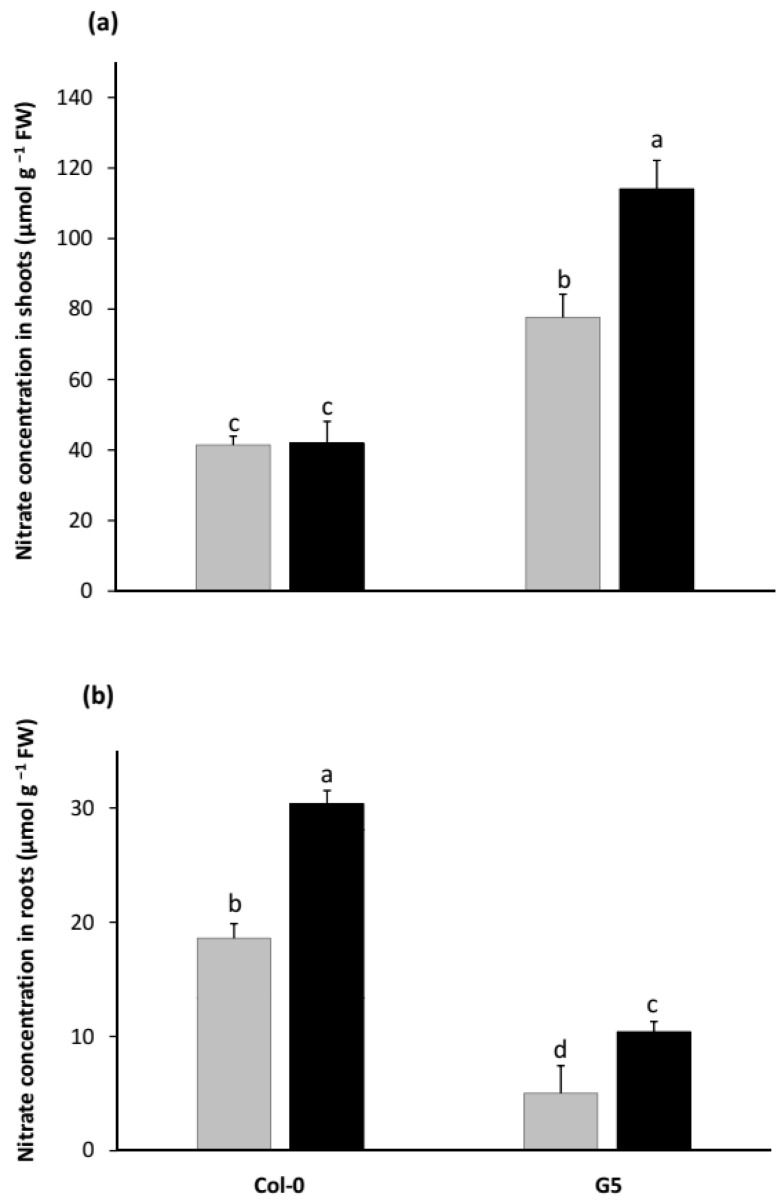
Effect of *nia2* mutation and inoculation with STM196 on NO_3_^−^ accumulation in shoots and roots of *Arabidopsis*. Col-0 and G5 mutant seedlings were grown on the NO_3_^−^ containing medium and transferred after 7 d on the same medium inoculated or not with 10^8^ cfu·mL^−1^ STM196, as described in Figure 1. (**a**) NO_3_^−^ content in shoots. (**b**) NO_3_^−^ content in roots. Grey bars, seedlings grown on uninoculated media; black bars, seedlings grown on media inoculated with STM196. Data are the means ± standard deviation (*n* = 20). Different letters represent significant differences using two-way ANOVA with Fisher’s LSD multiple comparison post-test at *p* = 0.001.

Since the shoot NO_3_^−^ the pool has been shown to be a good marker of the shoot N nutritional status that exerts an inhibitory effect on root growth in NO_3_^−^ grown plants [38], we used the shoot NO_3_^−^ content (Figure 3a) as a parameter to investigate the relationship between shoot NRA and root growth in seedlings inoculated (or not) with STM196.

As expected, decreased shoot NRA in G5 mutant led to increased NO_3_^−^ accumulation in shoots of axenically grown G5 compared to Col-0 seedlings (Figure 3a). Conversely, G5 mutants accumulated less NO_3_^−^ in their roots (Figure 3b) despite the decreased root biomass in comparison to Col-0 seedlings (Figure 2c). The difference in NO_3_^−^ root and shoot pools changes can be related to the very predominant distribution of nitrate reduction activity in shoots as mentioned above. Most of the NO_3_^−^ ions taken up by roots are thus likely to be translocated to the shoots where they are either accumulated or reduced to fuel the N assimilation pathway. The lack of NR activity in shoots would thus simultaneously lead to an increase in the size of leaf NO_3_^−^ pool and a decrease in amino acids synthesis rate. This, in turn, would lead to decreased shoot biomass production and amino acid translocation to roots and, hence, root biomass production.

Inoculation with STM196 triggered no change in the shoot NO_3_^−^ content of Col-0 seedlings whereas it induced a further increase in the shoot NO_3_^−^ content of G5 mutant seedlings (Figure 3a). While a negative correlation between shoot NO_3_^−^ content and root fresh weight was observed when considering the effect of the *nia2* mutation (G5 vs. Col-0, either non inoculated or inoculated), the changes in root biomass and shoot NO_3_^−^ concentration triggered by inoculation of either G5 or Col-0 with STM196 were not negatively correlated to each other (cf. Figure S2). Therefore, STM196 appears to alleviate the negative correlation between shoot NO_3_^−^ content and root biomass that has been well documented in axenically grown plants [27].

Consistently with previous data obtained in *Arabidopsis* seedlings grown in similar growth conditions [14], the amino acids found at the highest concentrations in our seedlings were GLU, GLN, ASP, ASN, SER, GLY, and ARG. To look for a possible change in N status downstream the nitrate uptake or reduction rates, we analyzed the levels of these seven amino acids in the shoots of seedlings uninoculated or inoculated with STM196 grown in the same NO_3_^−^ feeding conditions as before (Figure S3). The shoots of G5 seedlings had decreased levels in GLN, GLY, and ARG, while the levels of GLU, ASP, ASN, and SER were not substantially different from those of Col-0 seedlings. The inoculation with STM196 left the levels of free amino acids unchanged in the shoots of either the G5 mutant or Col-0 seedlings.

To further investigate the dependence (or independence) of STM196 triggered growth promotion over NRA or NO_3_^−^ ions accumulation in plant parts, we grew the Col-0, G5, and G′4-3 seedlings on an NH_4_^+^ containing culture medium (Figure 2b,d). One can note that Col-0 *Arabidopsis* seedlings fed with NH_4_^+^ displayed a lower growth rate and a lower shoot/root biomass ratio than those fed with NO_3_^−^ (4.0 vs. 4.7), a figure already described for *Arabidopsis thaliana* [39]. Under NH_4_^+^ nutrition conditions, G5 and G′4-3 mutant seedlings displayed similar shoot and root biomass production as the Col-0 seedlings, and the inoculation with STM196 induced shoot and root growth promotion to similar extents in the seedlings of the three lines (Figure 2b,d). These observations are consistent with the hypothesis that under NO_3_^−^ nutrition conditions the reduction of NRA does not affect STM196 induced growth promotion by itself, but hampers the ability of seedlings to produce more biomass as a consequence of N metabolism disorders.

### 2.2. nia Mutations and STM196 Inoculation Have Antagonistic Additive Effects on Root System Architecture

**Figure 4:**
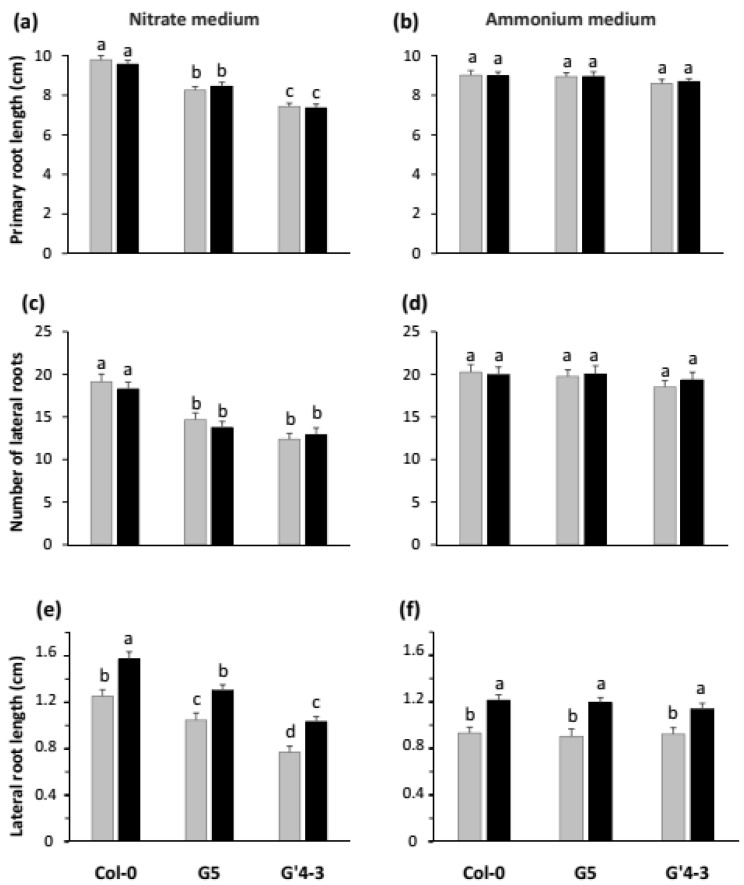
Effects of the *nia2* single mutation and *nia1 nia2* double mutation on root system architecture in *Arabidopsis*. Wild-type Col-0, G5, and G′4-3 mutant seedlings were grown on either nitrate or ammonium medium, and inoculated with STM196 (black bars) or not (grey bars) as described in Figure 2. The root system architecture was assessed from scanned images using ImageJ software, 7 d after seedlings transfer on the inoculated and uninoculated media. Following parameters were measured: the primary root length of seedlings grown on (**a**) the nitrate medium or (**b**) the ammonium medium; the number of lateral roots of seedlings grown on (**c**) the nitrate medium or (**d**) the ammonium medium; the average lateral root length of seedlings grown on (**e**) the nitrate medium or (**f**) the ammonium medium. Data are the means ± standard deviation (*n* = 20). Different letters represent significant differences using two-way ANOVA with Fisher’s LSD multiple comparison post-test at *p* = 0.001.

Under NO_3_^−^ nutrition conditions, the reduction of root biomass in axenically grown G5 and G′4-3 mutants (Figure 2c) was associated with a reduction in both the primary root length (Figure 4a) and the total length of lateral roots; this latter was due to both a significant reduction in the number of emerged lateral roots (Figure 4c) and the average length of lateral roots (Figure 4e). Consistently with previous observations on root biomass of NH_4_^+^ fed seedlings (Figure 2d), the *nia2* single mutation and *nia1 nia2* double mutation did not affect any root architecture parameters under NH_4_^+^ nutrition conditions (Figure 4b,d,f). This is not surprising since nitrate reductase activity is not involved in the nutrition of plants grown under NH_4_^+^ nutrition conditions.

As observed previously [26], the inoculation of NO_3_^−^ fed Col-0 seedlings with STM196 had no effect on either the primary root length or the number of lateral roots (Figure 4a,c), while it led to increased individual lateral root length (Figure 4e). These effects on root system architecture appear to be independent of those of the *nia* mutations since STM196 had similar effects on the three parameters analyzed in G5 and G′4-3 mutant seedlings and Col-0 seedlings (Figure 4a,c,e). Furthermore, the lateral roots of inoculated G5 and G′4-3 seedlings were slightly shorter than those of inoculated Col-0 seedlings, indicating an additivity of the negative effect of *nia* mutations and the positive effect of STM196 on lateral root growth. In addition, the lateral root length was also increased by inoculation with STM196 in all the three lines tested under NH_4_^+^ nutrition conditions, while the *nia* mutations had no effect on this parameter under these nutrition conditions (Figure 4b,d,f).

**Figure 5:**
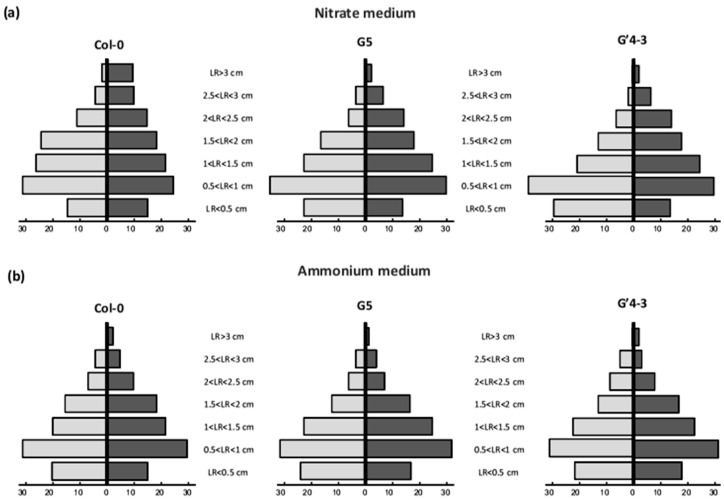
Response of lateral root growth rate to inoculation with STM196. *Arabidopsis* wild-type Col-0, G5, and G′4-3 mutant seedlings were grown and inoculated with STM196 as described in Figure 2, on (**a**) nitrate medium or (**b**) ammonium medium. The values presented are the percentages of lateral roots belonging to a given length class as indicated, calculated for 25–30 individual plants at 7 d after transfer onto uninoculated (pale grey bars, on the left side) or inoculated (dark grey bars, on the right side) media. LR: lateral roots.

Figure 4 shows that STM196 significantly increased lateral root length in G5 and G′4-3 mutants and wild-type seedlings, under NO_3_^−^ and NH_4_^+^ feeding conditions. To illustrate the impact of STM196 on the lateral root system, we designed another graph showing the distribution of lateral roots within classes of length (Figure 5). For any root system (uninoculated or inoculated by STM196), we measured the lengths of all visible lateral roots and attributed the roots to the corresponding length class. This alternative representation shows that the overall shape of lateral root distribution shifts to longer lateral roots in all these seedlings and N nutrition conditions. This result confirms our previous observation that STM196 triggers an increase in the relative proportion of longer lateral roots in NO_3_^−^ fed Col-0 [19,26,40]. Conversely, the *nia* mutations led to an increased proportion of shorter lateral root classes (<1 cm) and decreased sizes of longer lateral root classes (>2 cm) in NO_3_^−^ fed uninoculated seedlings (compare light grey bars in G5 and G′4-3 graphs to those in the Col-0 graph, Figure 5a). The inoculation with STM196 exerted the same effect in G5 and G′4-3 mutants as in the Col-0 seedlings: the proportion of longer roots (>2 cm) increased whereas the proportion of shorter roots (<1 cm) decreased in comparison with the uninoculated G5 and G′4-3 seedlings (compare dark grey to light grey bars within G5 and G′4-3 graphs, Figure 5a). Altogether, the distribution of the roots among length classes revealed the existence of antagonistic but additive effects of either the *nia* mutations or STM196 inoculation on lateral root growth of NO_3_^−^ fed seedlings: the architecture of the STM196-inoculated G5 and G′4-3 seedlings present an intermediate pattern between uninoculated G5 and G′4-3 seedlings and STM196-inoculated Col-0 seedlings. As expected from results in Figure 4, the *nia* mutations did not change the overall pattern of lateral root length distribution of NH_4_^+^ fed seedlings (Figure 5b). Under this nutrition condition, the inoculation with STM196 still increased the proportion of longer lateral roots and decreased the proportion of shorter lateral roots and, consequently, the overall pattern of lateral root length distribution of Col-0, G5, and G′4-3 STM196 inoculated seedlings are similar to each other. Overall, this analysis of lateral root length distribution of NO_3_^−^ or NH_4_^+^ fed, uninoculated or STM196 inoculated, Col-0, G5 and G′4-3 seedlings suggests that *nia* mutations and STM196 have antagonistic additive effects on root system architecture.

As previously shown [40,41], STM196 induced a strong elongation of roots hairs: under usual NO_3_^−^ nutrition conditions, the average root hair length was 0.25 mm in uninoculated plants and 0.84 mm in STM196 inoculated ones (Figure S4). The *nia2* mutation did not modify significantly root hair length and it did not hamper its three-fold increase under STM196 inoculation.

### 2.3. Inoculation with STM196 Have No or Very Low Effect on Nitrate Reductase Activity in Nrt2 Mutants

**Figure 6:**
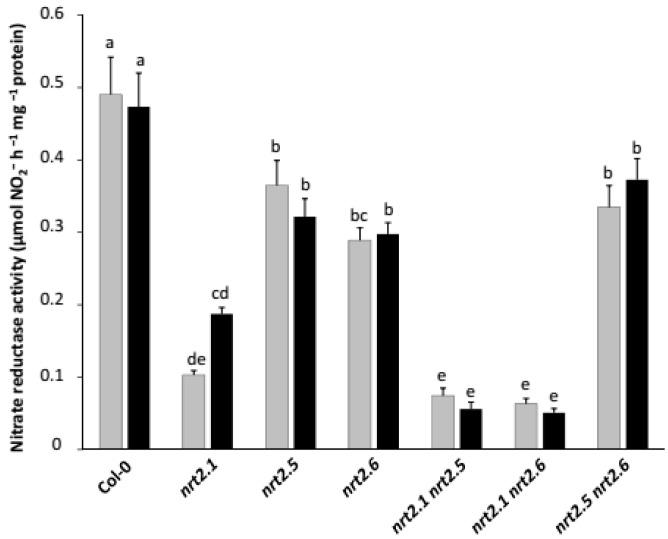
Effects of *nrt2.1*, *nrt2.5,* and *nrt2.6* single and double mutations on nitrate reductase activity. *Arabidopsis* seedlings of Col-0 (wild-type) and those of *nrt2.1*, *nrt2.5, nrt2.6*, *nrt2.1 nrt2.5*, *nrt2.1 nrt2.6* and *nrt2.5 nrt2.6* mutants were grown and inoculated as described in Figure 1. Eight days after the transfer onto STM196-inoculated (black bars) or uninoculated (grey bars) medium, the shoots were harvested and weighed, and the in vitro nitrate reductase activity was determined. Error bars indicate SD (*n* = 20−25 plants per condition). Different letters represent significant differences using two-way ANOVA with Fisher’s LSD multiple comparison post-test at *p* = 0.001.

NRA was not affected by STM196 inoculation either in Col-0 or G5 seedlings (Figure 1). To further investigate the effect of STM196 on NO_3_^−^ reduction, we analyzed NRA in mutant plants deficient in either the major root nitrate transporter, NRT2.1, or the two putative nitrate transporters required for plant growth promotion by STM196, NRT2.5, and NRT2.6. For this study, we used simple knock-out mutants *nrt2.1*, *nrt2.5,* and *nrt2.6* and the double mutants we generated as described in Kechid et al. [26]. Transcript measurements presented therein showed that double mutants were true loss-of-function mutants. Although there is some functional redundancy between several members of the NRT1 and NRT2 families of nitrate transporters, NRT2.1 has been shown to play a predominant role in NO_3_^−^ uptake by plants steadily supplied with moderate concentrations of NO_3_^−^ [42]. Compared to Col-0 seedlings, NRA was 3.5 fold lower in *nrt2.1* mutant seedlings (Figure 6). As expected in plants deficient for the major component of NO_3_^−^ uptake, NO_3_^−^ contents of both shoots and roots were also much reduced in the *nrt2.1* mutant [26]. Furthermore, in this later study, the decrease in shoot NO_3_^−^ concentration in *nrt2.1* mutant seedlings was not prevented by inoculation with STM196. The *nrt2.1 nrt2.5* and *nrt2.1 nrt2.6* double mutants showed no further diminution in NRA with comparison to single *nrt2.1* mutant (Figure 6). NRA was significantly lower in *nrt2.5* and *nrt2.6* single mutant seedlings than in WT seedlings (Figure 6). However, NRA levels did not differ between these two single mutant lines and the *nrt2.5 nrt2.6* double mutant line. Further, the impact of *nrt2.5* and *nrt2.6* mutations is very modest in comparison to the effect of *nrt2.1* mutation, which is consistent with the lack of effect of the *nrt2.5* and *nrt2.6* mutations in the *nrt2.1* background. Therefore, whereas NRT2.1 has a strong effect on NRA regulation, most probably because it controls the availability of NO_3_^−^ to nitrate reductase, the NRT2.5 and NRT2.6 proteins are unlikely to be essential for NRA. Remarkably, similarly to what was observed in Col-0 seedlings, the inoculation with STM196 did not affect NRA in any of the mutant lines analyzed (Figure 6).

### 2.4. Effects of nia2 Mutation and Inoculation with STM196 on Nitrate Reductase and Nitrite Reductase Genes Expression Levels

**Figure 7:**
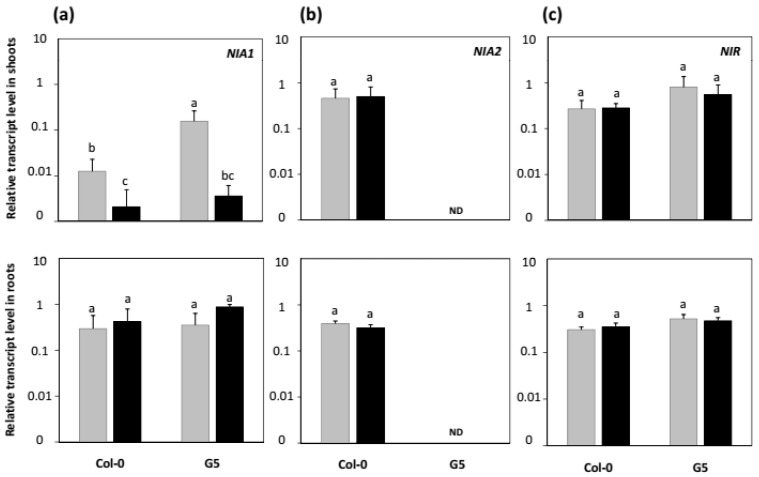
Effects of inoculation with STM196 on *NIA1*, *NIA2,* and *NIR* transcript levels. *Arabidopsis* seedlings of Col-0 and G5 mutant were grown and inoculated as described in Figure 1. Eight days after transfer on uninoculated (grey bars) or STM196-inoculated (black bars) medium, relative transcript levels of (**a**) *NIA1*, (**b**) *NIA2,* and (**c**) *NIR* were assessed in shoots (top panels) and roots (bottom panels) using quantitative real-time PCR analysis. The transcript levels were normalized against *Actin2* (AT3G18780) and *Ubiquitin* (AT4G05320) genes. Each bar represents the mean of at least three biological repetitions. Error bars indicate SD. Different letters represent significant differences using two-way ANOVA with Fisher’s LSD multiple comparison post-test at *p* = 0.001. nd: Not detected.

Consistent with previous reports [43], the *nia2* mutation induced a strong increase in *NIA1* expression in the shoots, but not the roots (*NIA1* transcript levels increased by more than 10-fold in shoots, Figure 7a). By contrast, the expression level of the nitrite reductase (*NIR*) gene was not significantly different in the roots and shoots of either G5 mutant or Col-0 seedlings (Figure 7c). In presence of STM196, there is a decrease in the expression of *NIA1* in shoots (Figure 7a). Interestingly, this downregulation of *NIA1* expression was visible in both Col-0 and G5 plants and, conversely, the increase in *NIA1* expression in *nia2* mutated seedlings did not seem to be alleviated by STM196 (Figure 7a). Altogether, the *NIA1* gene appeared to be regulated both positively in the *nia2* background (G5 mutant) and negatively by the STM196 rhizobacterium, but these regulations are seemingly independent of each other.

Inoculation of Col-0 seedlings with STM196 had no effect on the expression levels of genes coding for GS, GOGAT, or GDH isoforms (Figure S5). This result is consistent with data on amino acids accumulation, which showed no change in any of the major free amino acids accumulated in shoots (Figure S3).

**Figure 8:**
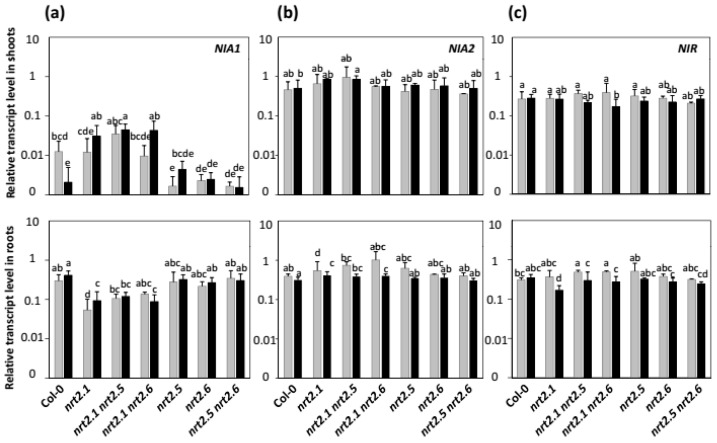
Effects of the *nrt2.1*, *nrt2.5,* and *nrt2.6* single and double mutations on *NIA1*, *NIA2,* and *NIR* transcript levels. *Arabidopsis* seedlings of Col-0 (wild-type) and *nrt2.1*, *nrt2.5*, *nrt2.6*, *nrt2.1 nrt2.5*, *nrt2.1 nrt2.6* and *nrt2.5 nrt2.6* mutants were grown and inoculated as described in Figure 1. Eight days after transfer on uninoculated (grey bars) or STM196-inoculated (black bars) medium, relative transcript levels of (**a**) *NIA1*, (**b**) *NIA2,* and (**c**) *NIR* were assessed in shoots (top panels) and roots (bottom panels) using quantitative real-time PCR analysis. The transcript levels were normalized against *Actin2* (AT3G18780) and *Ubiquitin* (AT4G05320) genes. Each bar represents the mean of at least three biological repetitions. Error bars indicate SD. Different letters represent significant differences using two-way ANOVA with Fisher’s LSD multiple comparison post-test at *p* = 0.001.

Since seedlings deficient in either the nitrate transporter NRT2.1 that plays a predominant role in NO_3_^−^ uptake or the NRT2.5 and NRT2.6 proteins that are key determinants of the growth promotion response to STM196 exhibited altered NRA (Figure 6), we investigated whether the related mutations affected the expression levels of the two *NIA* genes and the single *NIR* gene in *Arabidopsis*. Figure 8 shows that none of the *nrt2.1*, *nrt2.5,* and *nrt2.6* mutations triggered significant changes in the expression level of *NIA2* and *NIR* genes whether in roots or shoots. Moreover, this pattern is not modified by STM196 inoculation.

The expression of the *NIA1* gene was downregulated in the shoots, but not in the roots, of *nrt2.5* and *nrt2.6* single and double mutants (Figure 8a). This decrease in shoot *NIA1* expression was not related to any significant diminution of shoot NRA in *nrt2.5* and *nrt2.6* mutants (Figure 6). While STM196 inoculation resulted in a decrease in *NIA1* transcript level in the shoots of Col-0 seedlings, this transcript level slightly increased in those of *nrt2.1* mutant (Figure 8a). In the shoots of *nrt2.1 nrt2.5* and *nrt2.1 nrt2.6* double mutants, the *NIA1* transcript level was about the same as in the *nrt2.1* single mutant but higher than in the *nrt2.5* and *nrt2.6* simple mutants. This, again, shows that the *nrt2.1* mutation hides the effects of other mutations, most probably because the absence of the major NO_3_^−^ root transporter drastically impacts plant nutrition and growth capacity through a strong limitation in N provision under NO_3_^−^ nutrition conditions.

## 3. Discussion

The rhizobacterium STM196 promotes the growth rate of nitrate-grown *Arabidopsis* seedlings concomitantly with the enhancement of lateral root development [19,26]. The auxin-signaling pathway is involved in the lateral root response to STM196, but this strain does not produce, hence does not excrete, substantial amounts of auxin [19]. This previous study indicates that STM196 could trigger changes in endogenous IAA distribution independently from IAA released by the bacteria. Besides, two members of a nitrate-transporter gene family, namely *NRT2.5* and *NRT2.6*, are required for these responses [26], which may suggest some relation between plant growth promotion by STM196 and NO_3_^−^ transport in plants, in addition to a hormonal related phytostimulation effect. Interestingly, both *NRT2.5* and *NRT2.6* genes were also strongly upregulated in *Arabidopsis* treated with *Bacillus subtilis* strain L1 [12]. However, our previous results and those presented herein do not support a noticeable role of NRT2.5 and NRT2.6 either for NO_3_^−^ uptake or NO_3_^−^ distribution between roots and shoots in our growth conditions. This is in agreement with the report of an important role of NRT2.5 in NO_3_^−^ uptake by *Arabidopsis* plants only after a long period of starvation [29]. More precisely, Kotur et al. [44] showed that *NRT2.5* expression declined rapidly in the roots of nitrogen starved plants upon exposure to NO_3_^−^, so that 24 h after NO_3_^−^ resupply *NRT2.5* expression level in roots is much lower than in shoots. With regards to NRT2.6, up to now no nitrate-related phenotype has been observed in the *nrt2.6* knock-out mutant [30] suggesting a minor role, if any, in NO_3_^−^ uptake or distribution in the plant. Moreover, inoculation by STM196 did not significantly affect the expression of other members of the *NRT2* gene family which are known to be involved in NO_3_^−^ transport (Figure S6). By contrast, *NRT2.1* and *NRT2.2* were strongly overexpressed in seedlings treated with *B. subtilis* strain L1 for 4 d [12]. Since RNA was seemingly extracted from a whole seedling sample, where leaves are likely to represent the largest part of biomass, it has been hard to extrapolate upon the expression changes of *NRT2.1* and *NRT2.2* in roots specifically. However, considering that the root to shoot mRNA ratio for these two genes is in the 10 to 100 range [14], it is likely that *B. subtilis* strain L1 did increase *NRT2.1* and *NRT2.2* expression in roots, which could have led to the promotion of NO_3_^−^ uptake and, consequently, N nutrition. It is worth emphasizing the differences in plant culture conditions between the two studies: while we grew seedlings on a purely mineral medium, with no NH_4_^+^ source, and at physiological NO_3_^−^ concentration, in the *B. subtilis* study plants seedlings were grown on 2% sucrose-containing medium with an N source composed of both NH_4_^+^ and NO_3_^−^ and at a concentration of one order of magnitude higher than in our medium [12]. Thus, it is hard to speculate how *Arabidopsis* NO_3_^−^ transporter genes would respond to *B. subtilis* L1 inoculation in our culture conditions and to compare the results of the two studies. However, considering the large variety of effects that PGPR strains have on plants, it is likely that *B. subtilis* L1 and *P. brassicacearum* STM196 can differ in their mode of action whatever the culture conditions are.

To further investigate a possible role of NO_3_^−^ metabolism in the growth promotion response under NO_3_^−^ nutrition conditions, we used two nitrate reductase mutants, referred to as the G5 and G′4-3 mutants. The G5 mutant is a null mutant in the major nitrate reductase isoform since it has been shown to have a deletion of the entire *NIA2* gene [37]. In addition to the *NIA2* deletion, the G′4-3 mutant has a point mutation in the MoCo domain of the NIA1 protein causing severe disruption of NRA [36]. These single and double mutations inhibited shoot, and to a lesser extent, root growth under NO_3_^−^ nutrition. Contrary to our observation with G5 mutant, earlier reports mention an absence of the effect of single *nia2* mutation on plant growth [36,37]. This discrepancy is likely due to differences in cultural conditions. For instance, our seedlings have been grown on agar plates with NO_3_^−^ as the sole nitrogen source while the first G5 mutant analyses were performed on a soil mixture that could contain small amounts of other N forms. The ability of G′4-3 seedlings to grow, though at a strongly reduced rate, on NO_3_^−^ medium (Figure 2a) suggests that a low NRA remains in these seedlings, at least up to the young seedling stage analyzed in this study. This hypothesis is consistent with data published on the G′4-3 mutant, indicating that this mutant is strongly knocked-down but not devoid of NIA1 dependent NRA [36].

With regard to the possible involvement of NO_3_^−^ metabolism in the growth promotion response under NO_3_^−^ nutrition conditions, we did observe a perturbation of growth response of both roots and shoots in the G5 and G′4-3 mutants (Figure 2a,c). However, STM196 still enhanced lateral root development in the two mutants identically to what it did in Col-0 (Figure 4 and Figure 5), indicating that at least part of the developmental effect of STM196 is independent of NRA. Although G5 and even G′4-3 seedlings are not devoid of NRA, this hypothesis of independence of STM196 effect upon nitrate assimilatory pathway is more than likely when considering the low level of residual NRA in the G′4-3 mutant (below the detection limit, as mentioned above): lateral root response would not be identical in G′4-3 and the wild-type seedlings if NRA had a major role in this response. Remarkably, the reduction of lateral root elongation rate by the mutation in the *NIA2* gene and its increase in response to inoculation with STM196 are additive (Figure 4), as expected from two independent regulations. This conclusion is strengthened by the observation that STM196 induced increased root hair elongation in the G5 mutant similarly to what it did in WT plants (Figure S4), and thus independently to change in NO_3_^−^ metabolism.

Finely, the independence of STM196 growth-promoting effect and root system developmental changes vs. NO_3_^−^ reduction (NRA) and NO_3_^−^ pools is confirmed by the similarity of the effects triggered in Col-0 seedlings by the rhizobacterium under NO_3_^−^ and NH_4_^+^ nutrition conditions (Figure 2 and Figure 4). Furthermore, the *nia2* single mutation and *nia1 nia2* double mutation had no impact on growth and root developmental responses to STM196 under NH_4_^+^ nutrition conditions. Consistently with previous observations in similar culture conditions [14], inoculation with STM196 led to no change in major free amino acid pools in NO_3_^−^-fed G5 mutant as well as Col-0 plants (Figure S3),. This indicates that STM196 does not affect N metabolism downstream NO_3_^−^ uptake and reduction. Overall, the growth-promoting effect of STM196 is seemingly independent of the whole N assimilation pathway.

In a previous study [14], we showed that STM196 promotes plant growth independently from the level of NO_3_^−^ supply, thus alleviating N demand regulatory process that operates in nitrate-fed *Arabidopsis* under axenic conditions. In abiotic conditions, a negative correlation has been found between the accumulation of NO_3_^−^ in leaves and the root biomass [27]. In the present study, root fresh weight does seem to be negatively correlated with shoot NO_3_^−^ content (Figure S2). However, remarkably, the inoculation of Col-0 seedlings triggered an increase in root biomass but no change in NO_3_^−^ shoot pool, while the opposite trend was observed upon inoculation of the G5 mutant. This indicates that the negative correlation between root fresh weight and shoot NO_3_^−^ concentration mainly reflects the effect of the *nia2* mutation (G5 vs. Col-0), but not the impact of inoculation with STM196. Hence, in axenic conditions, a decrease in NRA did lead to increased NO_3_^−^ shoot pool and this was associated with decreased root growth rate as described earlier in tobacco [27], but root growth promotion by STM196 was not associated with changes in either NO_3_^−^ shoot pool or shoot NRA.

To explain that STM196 is unable to promote biomass production in G5 plants despite its ability to trigger the root developmental response, one should consider that nitrate reduction capacity is probably so low in G5 plants that N metabolism drastically limits biomass production. The developmental changes elicited by the rhizobacterium would then not lead to growth promotion because the growth rate potential does not allow any supplementary biomass production. In such a hypothesis, NRA catalyzed by the NIA2 isoform of nitrate reductase would be required for plant growth, but neither for the plant growth promotion effect of STM196 per se, nor for root development responses. Although this conclusion cannot be confronted directly in NO_3_^−^-fed seedlings, it is imposed by the similarity of the growth-promoting and root developmental responses to STM196 under NH_4_^+^ and NO_3_^−^ nutrition conditions (see discussion above).

The inoculation of Col-0 plants with STM196 did not change the *NIA2* expression level (Figure 7). Furthermore, STM196 did not affect the transcript accumulation of *NIR* gene and genes coding for GS, GOGAT, and GDH isoforms (Figure S5), indicating that the NO_3_^−^ assimilation genes are not targets of the signaling pathway(s) elicited by this rhizobacterium. Again, growth promotion by STM196 appears to be independent of NO_3_^−^ assimilation pathway. This contrasts with the recent study performed with *B. subtilis* strain L1 [12]: concomitant to a 4-fold increase in NRA discussed above, the level of *NIA2* transcripts was downregulated by *B. subtilis* L1. Again, this discrepancy can be due either to the differences in culture conditions or to the PGPR strains which can differ on their mode of action, and consequently on the response pathways they elicit in the plant.

The *NIA1* gene that codes for the second nitrate reductase isoform, was downregulated in inoculated seedlings of both Col-0 and G5 (Figure 7). This effect is additive to the *NIA1* upregulation in *nia2* mutant genetic background, suggesting independent controls. The changes in *NIA1* expression level do not affect NRA significantly, as shown by the steady level of NRA in response to inoculation with STM196. This result is consistent with previous reports indicating very minor participation of NIA1 to NO_3_^−^ reduction in *Arabidopsis* seedlings [36,37]. Remarkably, while STM196 downregulates *NIA1* expression specifically in the shoots, it does not in the roots (Figure 7). This result is to be related to a previous report that *NRT2.5* expression is higher in shoots than in roots of NO_3_^−^ grown plants [44] and, also, to our previous observations that both *NRT2.5* and *NRT2.6* expressions are higher in shoots [14,26]. This makes NRT2.5 and NRT2.6 more likely to act in the shoots, and not roots, to transduce the plant response to STM196. Furthermore, *nrt2.5* and *nrt2.6* mutations also repress strongly *NIA1* expression in the shoots, not roots (Figure 8), which is in favor of the involvement of the NIA1 protein in the NRT2.5 and NRT2.6 dependent signaling pathway of growth promotion. Our previous results on *NRT2.5* and *NRT2.6* and the results on *NIA1* presented herein both show that the effects of STM196 on root development and plant growth involve a systemic regulation with changes in leaf gene expression levels.

The inoculation with STM196 did not result in a modification of *NIA1* expression in *nrt2.5* and *nrt2.6* single and double mutants (Figure 8), suggesting that NIA1 acts downstream of NRT2.5 and NRT2.6. Contrary to what was observed in Col-0, STM196 led to *NIA1* overexpression in the shoots of *nrt2.1* mutants, even though the growth is strictly limited by NO_3_^−^ shortage in these plants. Again, the *NIA1* gene appears to respond in shoots to the signaling pathway elicited by STM196 independently from the control of NO_3_^−^ nutrition and plant growth potential.

Altogether, our results strongly suggest that NIA1 is involved in the NRT2.5 and NRT2.6 dependent signaling pathway elicited by STM196 we identified previously for *Arabidopsis* response to this PGPR strain [26]. Since we excluded the direct involvement of NO_3_^−^ transport and assimilation functions in the plant response to STM196, NIA1 is not likely to act in this response through a nitrate reductase activity to fuel N assimilatory metabolism, but through another activity. Besides its ability to reduce NO_3_^−^ in NO_2_^−^, NIA1 is also known to catalyze the production of NO [45,46], a signaling molecule known to be involved in the responses to various environmental stresses [47]. In our PGPR-plant interaction model, NIA1 could transduce the regulation exerted by NRT2.5 and NRT2.6 through modulation of NO production. Therefore, STM196 does not act as a biofertilizer that would improve N nutrition through nitrate reductase activity to fuel N metabolism. Instead, STM196 would act as photostimulation through an NRT2.5 and NRT2.6 signaling pathway, possibly involving NO production. In this hypothesis, STM196 would stimulate plant growth through development control pathways, but not via enhanced N assimilation and nutrition. Consistent with this hypothesis, we observed a limitation in plant growth promotion in NO_3_^−^-fed plants, as expected from N assimilate shortage, but not in NH_4_^+^-fed plants.

In *Arabidopsis*, the *NRT2.6* gene expression is also induced by the pathogenic bacterium *Erwinia amylovora* and it is correlated with better tolerance to pathogen attack [30]. Interestingly, the accumulation of H_2_O_2_ upon leaf inoculation with *E. amylovora* was lower in an *nrt2.6* knock-out mutant line and stronger in *NRT2.6* overexpressors as compared to the wild-type line, indicating a relation between NRT2.6 and reactive oxygen species (ROS) production in response to a biotic stress. Our results thus provide evidence in favor of the involvement of common regulatory elements, including ROS, in plant responses to either pathogen attacks or symbiotic interactions. NIA1 generated NO could be involved in this production of ROS in microorganism-challenged plants.

The effects of PGPR are very diverse [4,48,49,50] which suggests the involvement of a large diversity of signaling pathways in plants in which rhizosphere they colonize. Some PGPR could primarily enhance nutrition which would then lead to increased biomass production, whereas other PGPR would rather affect the development and growth rate of plants which will need a sufficient nutrient overall intake to sustain biomass production. In the former case, the rhizobacterium acts as a biofertilizer and in the latter case, it acts as a photostimulation. Data recently published [12] and our results herein suggest that *B. subtilis* L1 acts as a biofertilizer whereas STM196 acts as a photostimulation. Whereas the effect of STM196 has been shown to rely upon an NRT2.5–NRT2.6 dependent pathway [26], the effect of *B. subtilis* L1 is likely to be independent of this pathway.

## 4. Materials and Methods

### 4.1. Biological Material

All *Arabidopsis thaliana* (L.) Heynh lines used in this study are in the Col-0 ecotype background. Three single mutants affected in genes coding for nitrate transporters of the NRT2 family have been used: *nrt2.1-3* (SALK 035429) provided by Dr. Alain Gojon (BPMP, Montpellier, France), and *nrt2.5* (GK 213H10) and *nrt2.6* (SM 3.35179) supplied by Dr. Anne Krapp and Dr. Françoise Vedèle (IJPB, Versailles, France). Seeds of the single mutant *nia2* referred to as G5 [37], which is affected in the *NIA2* gene coding for the major nitrate reductase activity, have been provided by Dr. Alain Gojon. Seeds of the double mutant *nia1 nia2* referred to as G′4-3 [37], which is affected in the two nitrate reductase genes *NIA1* and *NIA2*, have been provided by Dr. Alain Gojon. Three double mutant lines, *nrt2.1 nrt2.5*, *nrt2.1 nrt2.6,* and *nrt2.5 nrt2.6*, have been generated in a previous study as described [26]. The *Phyllobacterium brassicacearum* STM196 strain [51] was chosen as a PGPR strain in this study for consistency with our previous work [26], and because it has been found in the rhizosphere of field-grown canola and efficiently promotes the growth of both canola and *Arabidopsis* [14,25].

### 4.2. Plant Growth Conditions and Inoculation

The *Arabidopsis* seeds were surface-sterilized through immersion in 0.57% sodium hypochlorite (*v*/*v*) supplemented with 0.095% Tween 20 (*v*/*v*) for 15 min. The seeds were washed five times in sterile distilled water and sawn in square Petri dishes (12 × 12 cm) onto solid 1.2% (*w*/*v*) agar (Sigma-Aldrich, St Louis, MO, USA) mineral medium where N was supplied as either 2 mM KNO_3_ [14] or 2 mM NH_4_Cl. Petri dishes were sealed with micropore tape^TM^ and stored at 4 °C in a dark room for 2 days. The Petri dishes were then placed vertically in a growth chamber under a regime of 16 h of light (130 µmol m^−2^ s^−1^, 22 °C) and 8 h of dark (18 °C) for 7 d. Thereafter, the seedlings were transplanted into new Petri dishes containing an identical fresh agar mineral medium inoculated or not with 10^8^ cfu·mL^−1^ of STM196 (see below). This device allows the root system of the seedlings to grow in direct contact with a medium homogeneously inoculated at a controlled bacterial concentration. The Petri dishes were aligned vertically for 8 additional days in the growth chamber.

The PGPR strain of STM196 was cultivated and grown on a solid medium 1.5% (*w*/*v*) agar containing mineral nutrients, 1% (*w*/*v*) mannitol, and 0.3% (*w*/*v*) yeast extracts (Sigma-Aldrich, St Louis, MO, USA) for 3 d at 25 °C (see E’ medium in Mantelin et al. [14]). To prepare the preculture, one single colony was isolated and inoculated into 20 mL of this liquid medium and incubated for 18 h at 25 °C on a rotary shaker (145 rpm). The final culture was obtained by inoculation of 2.6 10^9^ cfu·mL^−1^ from preculture to 500 mL of fresh liquid medium and incubated for 24 h under the same conditions as the preculture. To prepare the bacterial inoculum, the culture was pelleted by centrifugation at 5000× *g* for 5 min at room temperature. The pellet was resuspended in 20 mL of sterile liquid plant medium. The density of bacteria present in the inoculum was determined by the measurement of the absorbance at λ = 595 nm (WPA UV 1101, Biotech Photometer, Cambridge, UK). The STM196 inoculum was mixed with solid plant medium kept at 54 °C to a final concentration of 10^8^ cfu·mL^−1^ and poured into square Petri dishes for plant transfer (see above).

### 4.3. Root Architecture Analysis

The root system architecture was assessed at 7 d after seedlings transfer onto STM196-inoculated or non-inoculated medium, as previously described [19]. Briefly, numerical images of Petri dishes were recorded 7 d after transplantation using a flatbed scanner (Epson Perfection, V200, Epson, France) at a resolution of 400 dpi. Root system images were analyzed using the ImageJ software (Wayne Rasband, National Institutes of Health, Bethesda, MD, USA; http://rsbweb.nih.gov/ij/download.html, accessed on 27 December 2021), with the NeuronJ plugin (Erik Meijering, University Medical Center Rotterdam, NL; http://www.imagescience.org/meijering/software/neuronj/, accessed on 27 December 2021). Using this software, we measured the primary root length, the number of emerged lateral roots, and the length of each lateral root.

### 4.4. Measurements of Fresh Weight and Endogenous Nitrate Content

The shoots and roots of each seedling were harvested, separated, and weighed at 8 d after the transfer of the seedling on inoculated or non-inoculated medium. Nitrate was extracted from fresh tissues using 700 μL of 0.1M HCl at 4 °C for 48 h. Nitrate concentration in the extracts was measured using a continuous-flow automated analyzer (Autoanalyzer II Technicon, Tarrytown, NY, USA) through the reduction of nitrate to nitrite on a cadmium column and subsequent reactions with sulfanilamide and N-naphthyl ethylene diamine dichloride. The absorbance of the formed compound was measured spectrophotometrically at λ = 540 nm and compared with a nitrate standard curve to determine the nitrate concentration in each sample [52].

### 4.5. Measurements of Nitrate Reductase Activity

The shoots of each genotype inoculated or not with STM196 were harvested at 8 d after the transfer of the seedling on inoculated or non-inoculated medium. To this purpose, 500 to 600 mg of each fresh shoot sample was quickly frozen in liquid N_2_ and ground in a mixing mill (Retsch MM200, Retsch Hann, Germany). The frozen tissues were dissolved in 200 µL of the extraction buffer containing 80 mM K_2_HPO_4_, 20 mM KH_2_PO_4_, 1 mM EDTA, and 0.132% of cysteine, at pH 7.5. After centrifugation (10,000× *g*, 10 min, 4 °C), 10 µL aliquots were taken from the supernatants and preserved in new tubes for total protein measurement; the remaining supernatants were homogenized with 200 µL of an extraction buffer containing 2% (*w*/*v*) of BSA and centrifuged a second time. The nitrate reductase activity was then measured based upon Robin [53] and Mengel et al. [54]. The reaction was performed by adding the supernatant to a reaction mix containing 10 mM KNO_3_, 0.17 mM NADH, and 20 mM K_2_HPO_4_, 5 mM KH_2_PO_4_, and 0.05 mM EDTA buffered at pH 7.5, at 30 °C. The reaction was stopped after 30 min by the addition of 250 mM Zn acetate. After centrifugation at 10,000× *g* for 3 min, the quantity of nitrite (NO_2_^−^) formed was measured using sulfanilamide (0.05%, *w*/*v* in 1.5 N HCl) and N-naphthyl ethylene diamine dichlorure (0.01% *w*/*v*). After 15 min, the absorbance was measured at λ = 540 nm, and the NO_2_^−^ concentration of the biological samples was determined by comparison to a nitrite standard curve.

The total protein content was determined by the Bradford method [55]. The aliquot preserved from the first centrifugation after grinding was diluted 25 fold and 100 µL of these samples were homogenized with 900 µL of Bradford reagent (Sigma-Aldrich, St Louis, MO, USA). The absorbance was measured at λ = 595 nm and compared to a standard curve to assess the number of total proteins.

### 4.6. Analysis of Transcript Levels by Quantitative Real-Time PCR

Fifteen-day-old seedlings of the different genotypes, inoculated or not with the STM196 strain for the last 8 d, were harvested in the middle of the photoperiod, and shoots and roots were sampled separately. The samples were quickly frozen in liquid N_2_ and stored at −80 °C for a few days before extraction. Frozen tissues were ground in a mixing mill (Retsch MM200, Retsch, Hann, Germany), and total RNA was extracted using the SV Total RNA Isolation System (Promega, Madison, WI, USA) according to the manufacturer’s recommendations. The quantity of total RNA was assessed using UV spectrophotometry (Nanodrop N D1000, Thermo Fisher Scientific, Waltham, MA, USA).

1.5 µg of total RNA was treated with DNAse I (Invitrogen, Carlsbad, CA, USA), according to the manufacturer’s recommendations, for genomic DNA decontamination. First-strand cDNA was then synthesized using Super-Script II reverse transcriptase (Invitrogen, Carlsbad, CA, USA). The absence of genomic DNA contamination was verified through PCR using a primer pair designed across an intron spanning a region of the *Actin2* gene (At3g18780) (5′-ACTTTCATCAGCCGTTTTGA-3′ and 5′-ACGATTGGTTGAATATCATCAG-3′).

Relative gene expression was determined by quantitative real-time PCR on the MX3005P qPCR System (Agilent Technologies, Santa Clara, CA, USA). PCR was performed on 0.1 μL of first-strand cDNA with 0.5 µM of the primer pair of interest and 1X Brilliant II SYBR Green qPCR Master Mix (Agilent Technologies, Santa Clara, CA, USA) with the previously described cycles [26]. The following forward and reverse primers were used: *NIA1* (At1g77760), 5′-AGCGACGACGAAGACGAGAG-3′ and 5′-AATGGAGTGGAACTGGAGTGATG-3′; *NIA2* (At1g37130), 5′-CGATGTCCGAGGTCAAGAAGC-3′ and 5′-TTAGGCGAGGAAGAGTCAGAGG-3′; *NIR* (At2g15620), 5′-TGGTGTTGTGTTGCCTGATGTG-3′ and 5′-TTAGCGGTGATAAACTGCGAAAGG-3′. To determine the relative abundance of the transcripts, all qPCR results were standardized to two constitutive genes, *Actin2* (At3g18780) and *Ubiquitin10* (At4g05320), using gene-specific primers (*Actin2* forward 5′-TCCCTCAGCACATTCCAGCAGAT-3′ and *Actin2* reverse 5′-AACGATTCCTGGACCTGCCTCATC-3′; *Ubiquitin10* forward 5′-CACACTCCACTTGGTCTTGCGT-3′ and *Ubiquitin10* reverse 5′-TGGTCTTTCCGGTGAGAGTCTTCA-3′).

### 4.7. Statistical Analyses

Each experiment was repeated independently at least three times. Fresh weights, NO_3_^−^ accumulation, and nitrate reductase activity data as well as root system architecture measurements and relative transcript levels were analyzed using XLSTAT version 2010. Data were analyzed by two-way ANOVA and the significance between the means of different treatments was evaluated using a Fisher’s LSD test at *p* ≤ 0.001.

## Data Availability

Not applicable.

## Supplementary Materials

The following supporting information can be downloaded at: https://www.mdpi.com/article/10.3390/plants11010128/s1, Figure S1: Typical images of the culture device; Figure S2: Relationships between the fresh weight or nitrate reductase activity, and the NO_3_^−^ content; Figure S3: Shoot main free amino acid contents in nitrate-fed *Arabidopsis* seedlings; Figure S4: Effect of the *nia2* mutation on root hairs length; Figure S5: Relative transcript levels of genes coding for GS, GOGAT and GDH isoforms in nitrate-fed *Arabidopsis* Col-0 seedlings. Figure S6: Transcript levels of *NRT2* genes family in the *nia2* single mutant and *nrt2.5 nrt2.6* double mutant.
