# Supplementary material for: Arabidopsis Growth-Promotion and Root Architecture Responses to the Beneficial Rhizobacterium Phyllobacterium brassicacearum Strain STM196 Are Independent of the Nitrate Assimilatory Pathway
Source: Plants (Basel). 2022 Jan 4;11(1):128. doi: 10.3390/plants11010128 (PMC8747170; doi:10.3390/plants11010128)
Supplement: Supplementary file 1 [file plants-11-00128-s001.zip › plants-1443698-supplementary.pdf]

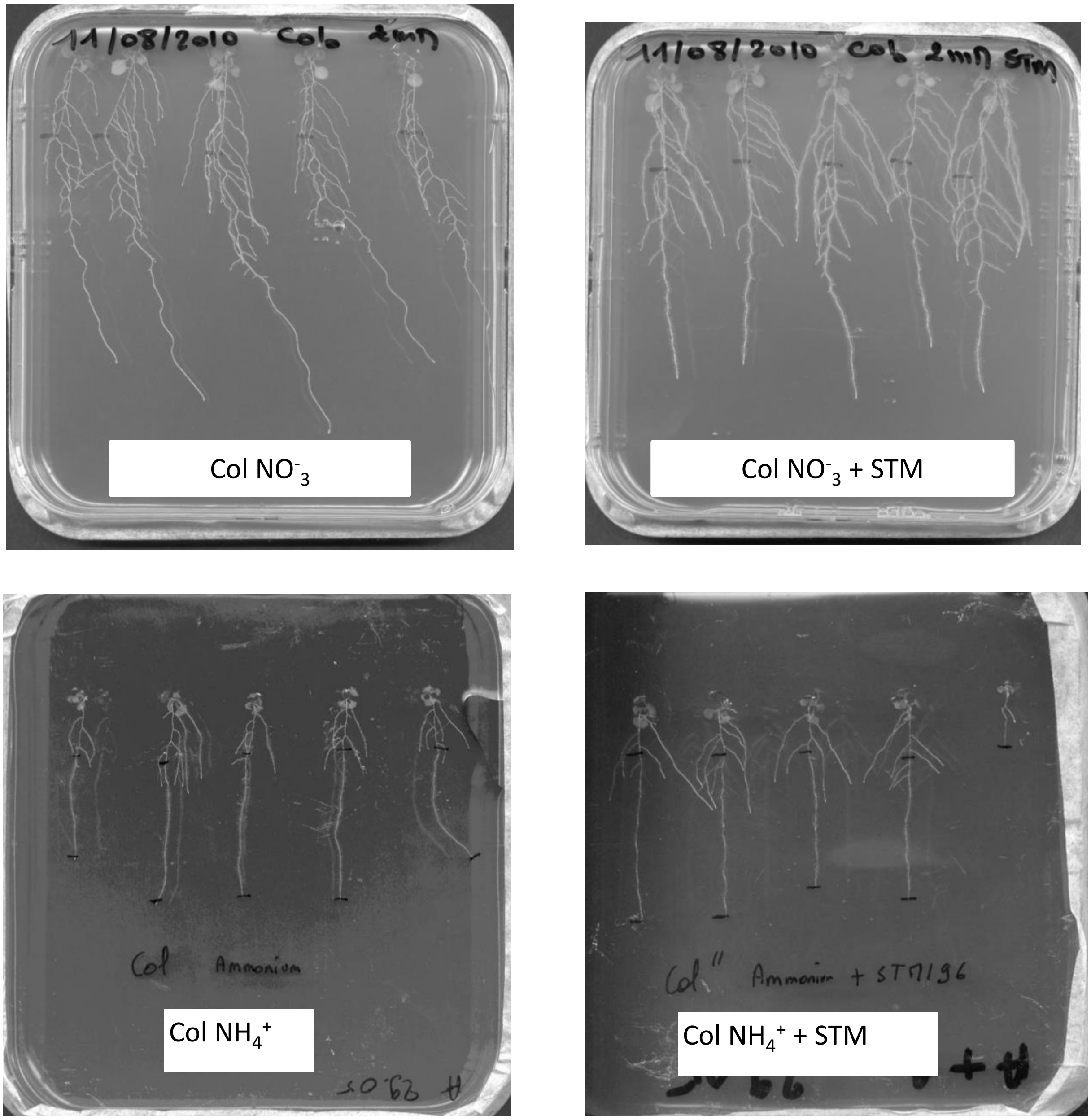

Figure S1. Typical images of the culture device. The *Arabidopsis* seeds were surface-sterilized and sawn in square Petri dishes (12 x 12 cm) onto solid 1.2% (w/v) agar mineral medium where N was supplied as either 2 mM KNO_3 or NH_4Cl 2 mM. After a storage at 4°C in a dark room for 2 days, the Petri dishes were placed vertically in a growth chamber under a regime of 16h of light (130 μmol m^{-2} s^{-1}, 22°C) and 8h of dark (18°C) for 7 d. Thereafter, the seedlings were transplanted into new Petri dishes containing an identical fresh agar mineral medium inoculated or not with 10^8 cfu mL^{-1} of *P. brassicacearum* STM196. This device allows the root system of the seedlings to grow in direct contact with a medium homogeneously inoculated at a controlled bacterial concentration. The Petri dishes were aligned vertically for 8 additional days in the growth chamber. Shown are Petri dishes of wild-type plants grown on either NO_3^- or NH_4^+ medium, inoculated with STM196 or not, as indicated.

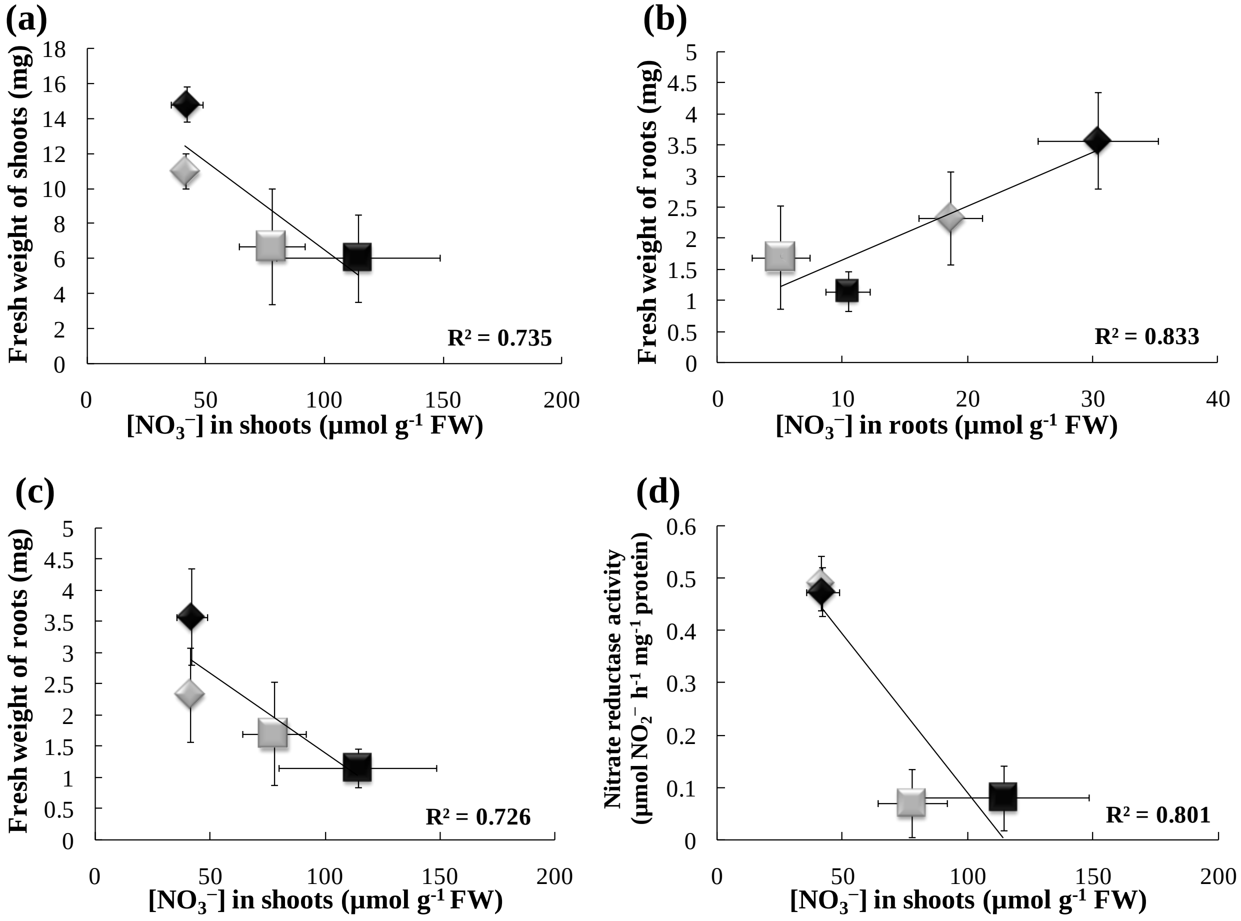

Figure S2. Relationships between the fresh weight or nitrate reductase activity, and the NO_3^-content. *Arabidopsis* Col-0 (diamonds) and G5 (squares) mutant seedlings were grown as described in Materials and Methods. Shoots and roots were harvested separately and weighed 8 days after the transfer on uninoculated (grey) or STM196-inoculated (black) medium. Nitrate content was assessed in both shoots and roots, while detectable nitrate reductase activity was measured only in shoots. (a), relationship between shoot biomass and NO_3^-concentration in shoots; (b), relationship between root biomass and NO_3^-concentration in roots; (c), relationship between root biomass and NO_3^-concentration in shoots; (d), relationship between shoot nitrate reductase activity and NO_3^-concentration in shoots. Error bars indicate SD (n = 20).

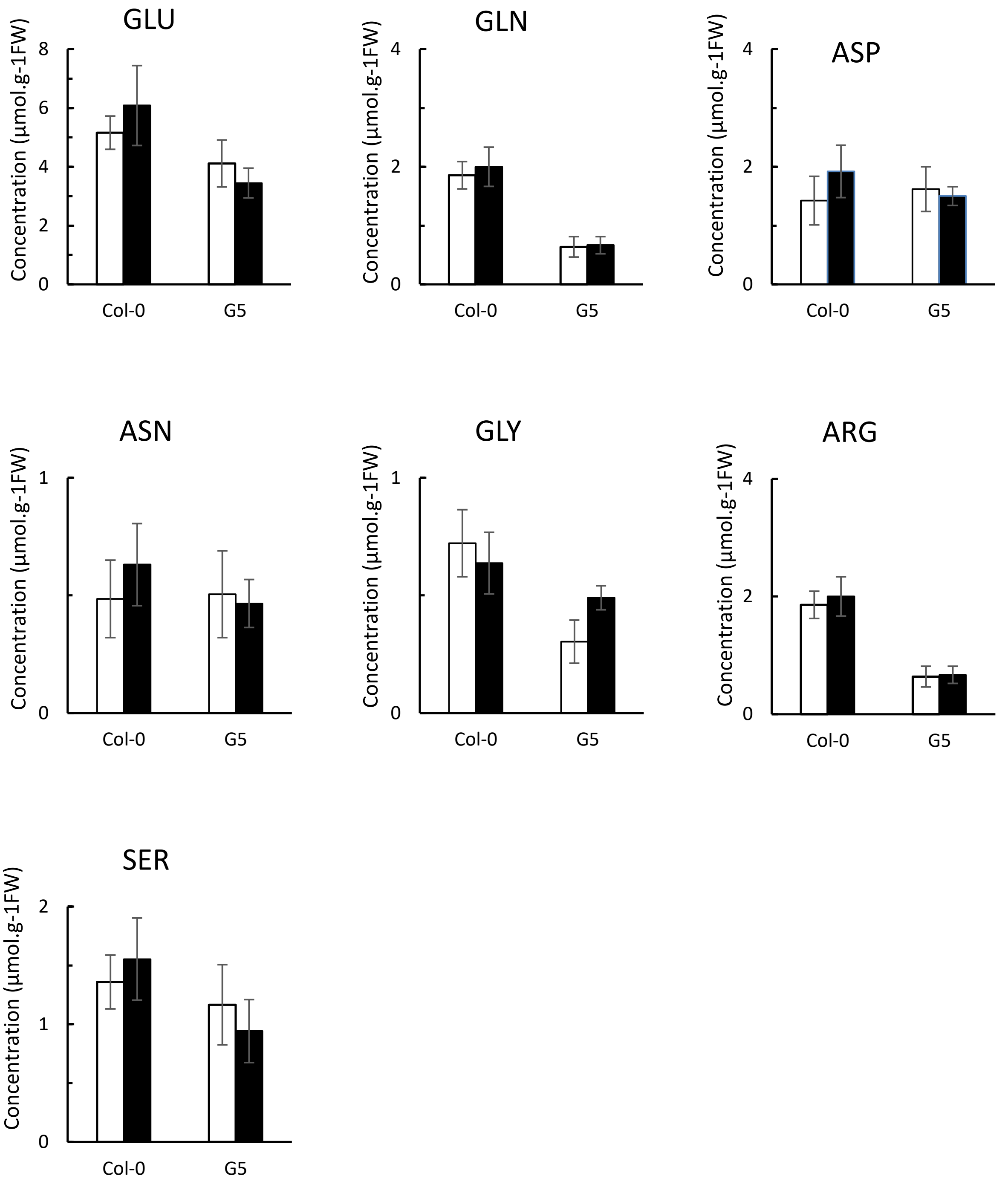

Figure S3. Shoot main free amino acid contents in nitrate-fed Arabidopsis seedlings. The seedlings were grown in vertically-oriented Petri dishes, on an agar mineral medium where N was supplied as 2 mM KNO_3 , as described in Materials and Methods. Eight days after transfer on fresh uninoculated (white bars) or STM196-inoculated (black bars) medium, amino acids were extracted from fresh shoot tissues at 4°C, and assayed by HPLC as described in Muller and Touraine (1992, J. Exp. Bot., 43, 617-623). Error bars indicate SD (n=10). No significant difference was found between the amino acid contents of uninoculated vs STM19-inoculated seedlings, for either the Col-0 wild-type ecotype or G5 mutant seedlings.

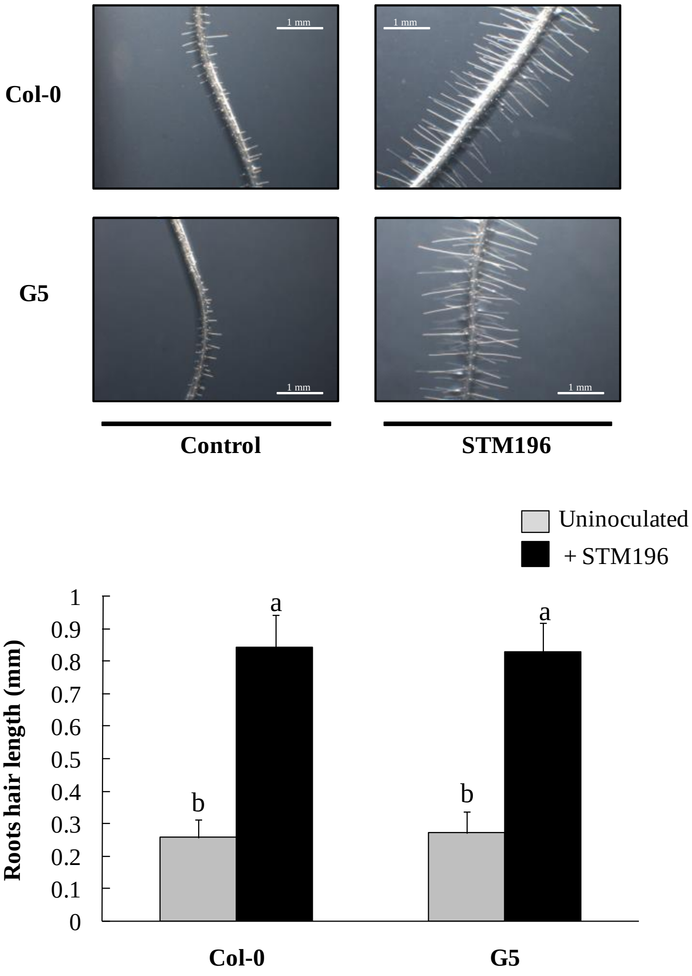

Figure S4. Effect of the *nia2* mutation on root hairs length. *Arabidopsis* Col-0 and G5 mutant seedlings were grown and inoculated (black) or not (grey) as described in the Materials and Methods. The length of root hairs was measured 7 days after seedlings transfer on fresh uninoculated or STM196-inoculated medium, using a macroscope (Z16APO, Leica, Bensheim, Germany). Error bars indicate SD (n = 20-25 plants per condition). Different letters represent significant difference using two-way ANOVA with Fisher's LSD multiple comparison post-test at P = 0.001.

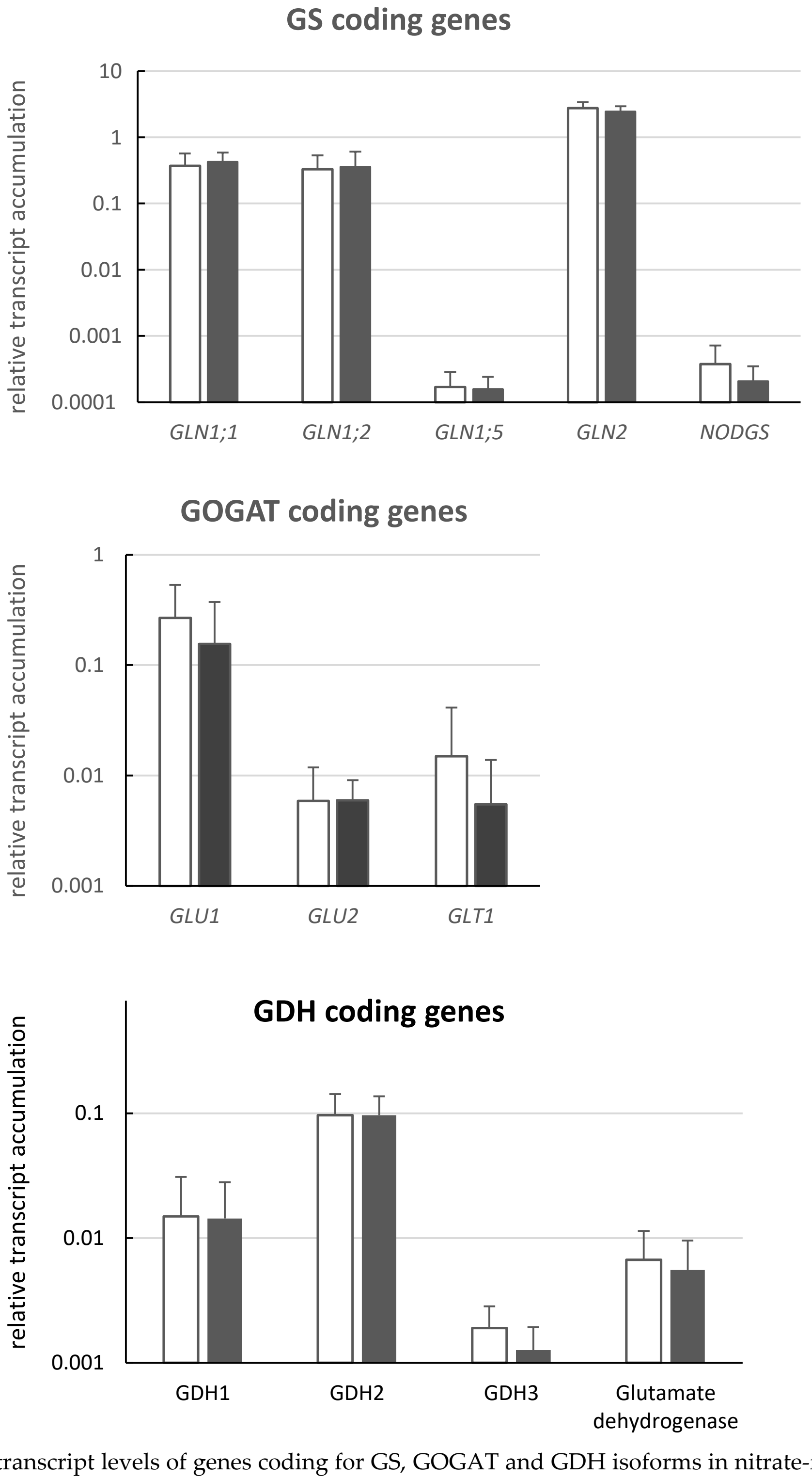

Figure S5. Relative transcript levels of genes coding for GS, GOGAT and GDH isoforms in nitrate-fed *Arabidopsis* Col-0 seedlings. The seedlings were grown in vertically oriented Petri dishes, on an agar mineral medium where N was supplied as 2 mM KNO3, as described in Materials and Methods. Eight days after transfer on fresh uninoculated (white bars) or STM196-inoculated (black bars) medium, relative transcript levels of GS, GOGAT and GDH coding genes were assessed in shoots using quantitative real time PCR analysis as described in Materials and Methods. GLN1;1, At5g37600; GLN1;2, At1g66200; GLN1;5, At1g48470; GLN2, At5g35630; NODGS, At3g53180; GLU1, At5g04140; GLU2, At2g41220; GLT1, At5g53460; GDH1, At5g18170; GDH2, At5g07440; GDH3, At3g03910; Glutamate dehydrogenase, At1g51720. The transcript levels were normalized against Actin2 (At3g18780) and Ubiquitin (At4g05320) genes. Each bar represents the mean of at least three biological repetitions. Error bars indicate SD. For all the genes tested, no significant difference in transcript relative accumulation was seen between the shoots of uninoculated and STM196-inoculated seedlings.

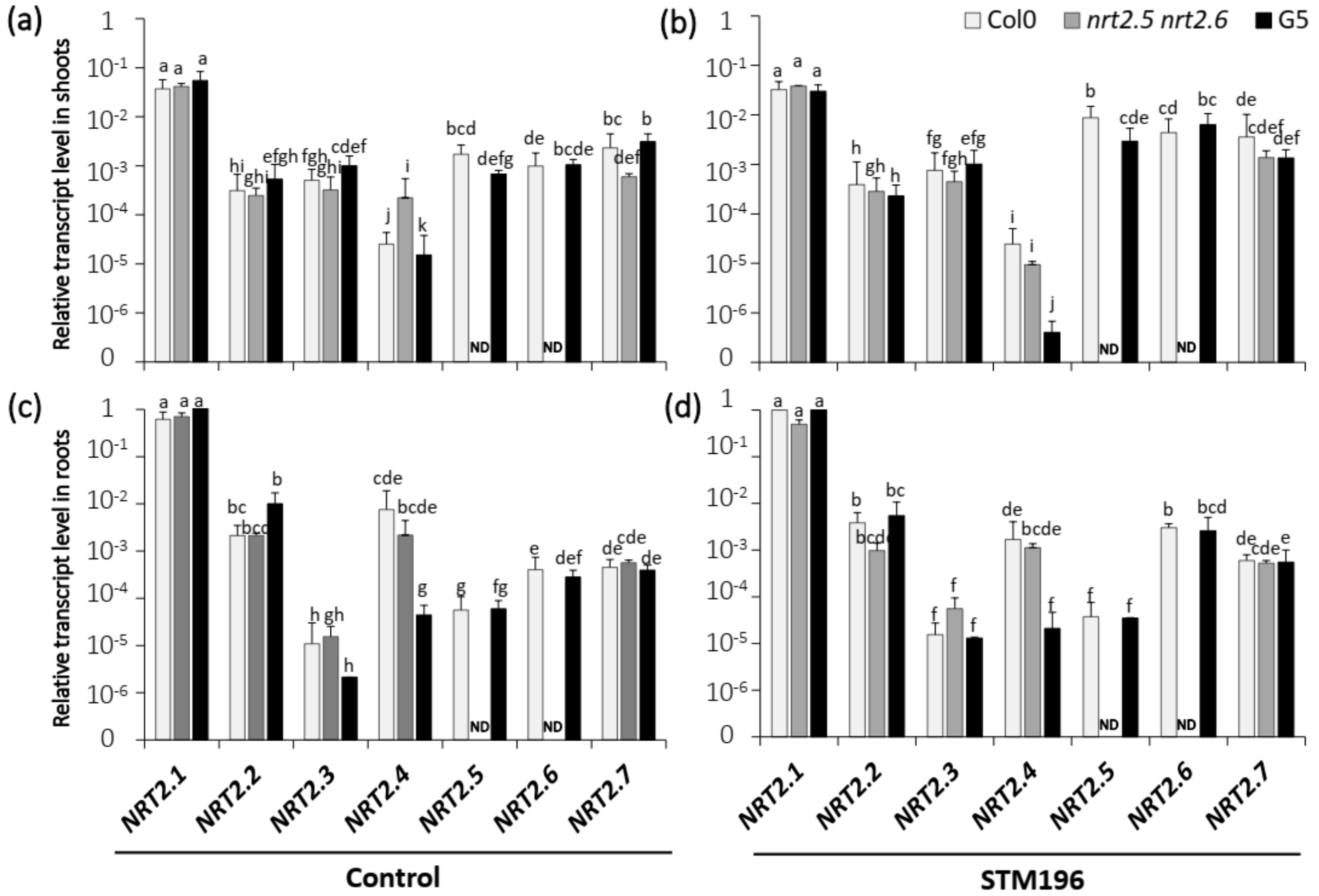

Figure S6. Transcript levels of *NRT2* genes family in the *nia2* single mutant and *nrt2.5 nrt2.6* double mutant. *Arabidopsis* seedlings of Col-0 (wild-type), G5 mutant and *nrt2.5 nrt2.6* double mutant were grown and inoculated as described in Materials and Methods. Eight days after transfer on fresh uninoculated or STM196-inoculated medium, relative transcript levels of *NRT2* genes family were assessed in shoots and roots using quantitative real time PCR analysis as described in Materials and Methods. (a) Shoots, uninoculated seedlings; (b) Shoots, STM196-inoculated seedlings; (c) Roots, uninoculated seedlings; (d) Roots, STM196-inoculated seedlings. The transcript levels were normalized against *Actin2* (AT3G18780) and *Ubiquitin* (AT4G05320) genes. Each bar represents the mean of at least three biological repetitions. Error bars indicate SD. Different letters represent significant difference using two-way ANOVA with Fisher's LSD multiple comparison post-test at P = 0.001. ND: Not detected.
